#  Potential of Drug Interactions among Hospitalized Cancer Patients in a Developing Country 

**Published:** 2013

**Authors:** Maria Tavakoli-Ardakani, Kaveh Kazemian, Jamshid Salamzadeh, Mahshid Mehdizadeh

**Affiliations:** a *Department of Clinical Pharmacy, School of Pharmacy, Shahid Beheshti University of Medical Sciences, Tehran, Iran. *; b *Pharmaceutical Sciences Research Center, Shahid Beheshti University Of Medical Sciences, Tehran, Iran. *; c *Department of Clinical Pharmacy, School of Pharmacy, Mazandaran University of Medical Sciences, Sari, Iran. *; d *Bone Marrow Transplantion Center, Taleghani Hospital,Shahid Beheshti University Of Medical Sciences, Tehran, Iran.*; e *Students Research Committee, Shahid Beheshti University of Medical Sciences, Tehran, Iran. *

**Keywords:** Interactions, Hospitalized cancer patients, Potential

## Abstract

Cancer patients are more susceptible to adverse drug-drug interactions (DDIs) due to receiving multiple medications especially chemotherapy medications, hormonal agents and supportive care drugs. The aim of this study is to describe the prevalence of potential DDIs and to identify risk factors for these potential interactions in hospitalized cancer patients in a developing country.

A cross-sectional study conducted by reviewing charts of 224 consecutive in hospitalized patients in hematology-oncology ward of a teaching hospital in Tehran, during a 12 month period from July 2009 to July 2010. “Drug Interaction Facts 2008, 2009: The Authority on Drug Interactions” was used for screening the potential drug-drug interactions. Potential interactions were classified by levels of severity and documentation.

The median age of patients was 50 years, the length of hospital stay for patient was 5 days and the number of drugs per patient was 8 drugs. Two hundred and twenty-eight potential interactions were detected. Nearly 14% of the interactions were major and 60% were moderate. Approximately 9% and 10% potential interactions were graded as established and probable. In multivariate analysis, being older than 61 years old, suffering from hematologic cancer, source of cancer in different specific organs (esophagus, testis and cervices more than other sources), and number of ordered drugs for patients were independent predictors of having at least one potential DDI in hospital order. Suffering from hematologic cancer, source of cancer in different organs, length of hospital stay and number of ordered drugs for patients were independent predictors for number of interactions per patients.

Having a DDI seems to be more likely to occur in patients older than 61 years old. Hematologic cancers, having more medications in physician’s order, longer length of hospital stay, esophageal cancer, testicular cancer and cervical cancer have related to having a DDI and also having more number of interactions.

## Introduction

Drug-Drug Interactions (DDIs) cover nearly 20-30% of adverse drug reactions. In elderly patients, it is increased to 80% and some of these interactions may become irreversible adverse reactions and basic health damage. (1) Receiving multiple medications, cancer patients are more susceptible to adverse drug-drug interactions. Beside their cancer pharmacotherapy include cytotoxic chemotherapy medications, hormonal agents and supportive care drugs, these patients are often elderly and use medications require for co-morbid conditions such as rheumatologic, gastrointestinal and cardiovascular disease (2). Cancer patients are particularly more susceptible to pharmacokinetic parameters alteration since they often suffer from mucositis, malnutrition, generalized edema and reduction of serum-binding proteins, hepatic and renal function which always heightened in elderly patients. This alteration in absorption, distribution, metabolism and exertion can also increase the risk of DDIs and promote them to an important cause of morbidity and mortality in cancer patients. It is showed that 4% of mortality in oncology ward is due to the drug interactions (1).

Pharmacokinetic, pharmacodynamic and pharmaceutical are three types of drug interactions (2). Mechanisms of the pharmacokinetic interactions were related to metabolism and/or exertion, absorption, elimination and distribution (3). When Allopurinol and Mercaptopurine are administered together, a Pharmacokinetic DDI happens since Allopurinol alters the metabolism of Mercaptopurine (4). Pharmacokinetic DDI is a kind of interaction in which one drug changes the pharmacokinetic factors of another drug (absorption, distribution, metabolism and/or exertion). When Fluorouracil and Warfarin are administered together, a pharmacodynamic DDI happens as Fluorouracil may significantly potentiate the hypoprothrombinemic effect of Warfarin (5). Pharmacodynamic DDI is a kind of interaction that happens between two drugs at the site of action. When Penicillins and Aminoglycosides are administered together, a pharmacodynamic DDI happens as they inactivate each other in same IV. solution (6). Pharmaceutical DDI is a kind of interaction in which a physical or chemical incompatibility in intravenous injections exists between two different drugs.

In these years, several studies have investigated the potential for drug interactions in cancer patients in developed countries (7-14). In a Canadian study (2007) between 4 previous medications of out-patient receiving systemic cancer therapy for solid tumor, potential drug interaction associated with increasing number of medication of patients, type of medications (medications require for co-morbid conditions) and presence of brain tumor (10). But the potential of this action in cancer patients of developing countries is largely unknown. In a Brazilian study (2005) potential for drug interactions unrelated to chemotherapy in hospitalized cancer patients found to be 63% in the day half-way through the hospital stay but this study didn’t investigated all patients medication (15). Propose of this study is to describe the prevalence of potential DDIs regardless of whether they actually occurred clinically. Also we want to correlate the frequency of DDIs with demographic information of patient and recognize risk factors for these potential DDIs in a hematology-oncology ward of hospitalized patients in a developing country, Iran.

## Experimental

*Methods*

A cross-sectional study was conducted on 224 consecutive in-hospital patients in hematology-oncology ward of Taleghani hospital located in Tehran, during a 12 mounth period from July 2009 to July 2010. Taleghani Hospital is affiliated to Shahid Beheshti University of Medical Sciences and is one of the largest referral cancer centers in Iran.

Our study assessed hospitalized patients’ medical chart records on hematology-oncology ward. All hospitalized patients in hematology-oncology ward with diagnosis of solid tumor and hematologic malignancy and were currently receiving standard systemic cancer-directed treatment were considered to be eligible.

In this ward, patients were visited 2 times a day by their physicians. After visiting and ordering registration in chart records, each order was tabulated in a designed form. These forms have demographic and medical information of patients and were filled 2 times a day for each patient. All ordered drugs in patients chart records were tabulated in the forms for analyze regardless of their actual administration. Complementary, alternative and herbal medication and foods of patients did not tabulate in designed forms for future analyze.

Since the original software for drug interactions was not available, “Drug Interaction Facts 2008, 2009: The Authority on Drug Interactions” was used for screening the potential DDIs (16, 17). This book has classified the potential DDIs by severity and documentation levels. By the level of severity, potential DDIs have been scored to three level of major, moderate and minor (Table 1). By the level of documentation they have been scored to five level of established, probable, suspect, possible and unlikely (Table 2).

**Table 1:** Drug-Drug interactions by severity levels (16, 17).

**Severity levels**	**Potential effect of an adverse effect**
Major	Life-threatening or permanent damage
Moderate	Deterioration of patient’s status
Minor	Bothersome or little effect

**Table 2:** Drug-Drug interactions by documentation level (16, 17).

**Documentation level**	**Type of scientific data for adverse effects**
Established	Proven to occur in well-controlled studies
Probable	Very likely, but not proven clinically
Suspected	May occur; some good data, but needs more study
Possible	Could occur, but data are very limited
Unlikely	Doubtful; not good evidence of a clinical effect

Descriptive statistics including central tendency measures and variability measures were used to describe the data. Kolmogorov-Smirnov test was used to evaluate the normal distribution of the variables. Independent sample t-test, One-way Analysis of Variance, and chi-square test were applied to assess differences among groups, where appropriate. Pearson correlation coefficient was used to assess the relationship between quantitative variables. Multivariate linear and logistic regression was applied with to predict dependent variables including age, gender, type of cancer, source of cancer, number of ordered drugs and length of hospital stay based on independent variables including having at least one potential DDI on the phamacotherapeutic protocol, and number of potential DDIs per patients. Odds ratios and their 95% confidence intervals (CI) were estimated through logistic regression model. Probability value of less than 0.05 was considered significant.

## Results

For one year period from July 2009 to July 2010, chart records’ drugs orders of 224 consecutive eligible cancer hospitalized patients were studied. Patients’ characteristics are shown in Table 3. 

**Table 3:** Patients’ characteristics

**Variable**	**Range Median**	**Number of patients**
Age (years)	14-90 50	
Sex		
Female		84 (37.5%)
Male		140 (62.5%)
Tumor		
Solid		163 (28%)
Hematologic		61 (72%)
Length of hospital stay (days)	2-40 4	
Number of drugs per patient	2-24 8	

Median age of patients was 50 years ranging from 14 to 90 years. Females were 37.5% of patients and approximately 73% of patients were diagnosed with a solid tumor. Length of hospital stay for patients varied from 2 to 40 days, with a median of 5 days. Number of drugs in patients order ranged from 2 to 24 drugs, with a median of 8 drugs.

Number of potential DDIs found for each patient in the ward ranged from 0-19 interactions and the mean of potential DDI for each patient is 1.02. All potential for these 224 patients were 228 interaction in this one year and 84 patients (37.5%, 95%CI: 31-44%) had at least one potential interaction on their screening.

Severity and documentation levels of potential DDIs are shown on Tables 4 and 5 . According to the severity level, 14.03% of interactions were classified as major and 59.65% of them classified as moderate. Based on the documentation level, 19.29% of the interactions classified as established and probable.

**Table 4:** Drug-drug interactions by severity levels on patients found by screening

**Severity levels**	**Frequency**	**Percentile**
Major	32	14.03%
Moderate	136	59.65%
Minor	60	26.31%

**Table 5:** Drug-drug interactions by documentation level

**Documentation level**	**Frequency**	**Percentile**
Established	21	9.21%
Probable	23	10.09%
Suspect	68	29.82%
Possible	95	41.67%
Unlikely	21	9.21%

A qualitative study was done on the screened potential DDIs. The most frequent combination with the potential of interaction in 224 patients was Cisplatin with Furosemide which encountered 19 times. Next potential interaction found was Magnesium hydroxide with Dexamethasone which encountered 7 times. Potential interaction of Magnesium hydroxide with Ranitidine encountered 6 times. Potential interactions between Ciprofloxacin with Doxorubicin and Acetaminophen with Furosemide had the next frequency by 5 times.

Table 6 demonstrates characteristics of the two groups of patient: patients with no potential drug interaction and patients which have at least one potential interaction on their orders. Patients with at least one potential interaction are likely to be older than 61 years old than patients with no potential drug interaction (p = 0.024). But no significant association explored between patients older than 61 years old and number of interactions and also no correlation was evident between number of interactions in patients and age (r = - 0.05, p = 0.938). Gender has no significant tendency on two groups of patients, with and without potential interaction, and also gender did not affect number of potential DDIs per patients. In patient with hematologic cancer having potential DDI was significantly more comparing with patients with a solid tumor (54.09% versus 31.28%) (p = 0.002) and also number of interactions in patients with hematologic cancer is significantly more than number of interactions in patients with solid tumor (p = 0.019). Number of ordered drugs and longer length of hospital stay were associated with having potential DDI in patients (p < 0.001, p < 0.001). Also both number of the ordered drugs and longer length of hospital stay were correlated with the number of interactions found for each patients (r = 0.601 and 0.806, p < 0.001 and p < 0.001). Source of cancer in different organs have also associated with having potential DDI in patients (p < 0.001). All patients with esophageal cancer, testicular cancer and cervical cancer have at least one interaction on their medications. So these patients are more probable to have interactions. Also number of interactions per patient depends on the source of cancer. (p = 0.007).

**Table 6:** Characteristics of the two groups of patients: patients with no potential drug interaction and patients which have at least one potential interaction on their orders and their univariate analysis

**Variable**	**Total**	**With interaction**	**Without interaction**	**p-value**
Age (years)	48.39 ± 18.39	51.12 ± 19.72	46.75 ± 17.42	0.18
Sex (n, %)				
Male	140, 62.5%	54, 64.3%	86, 61.4%	0.66
Female	84, 37.5%	30, 35.7%	54, 38.6%	
Tumor (n, %)				
Solid	163, 72.8%	51, 60.7%	112, 80%	0.002
Hematologic	61, 27.2%	33, 39.3%	28, 20%	
Length of hospital stay	6.54 ± 5.45	8.55 ± 7.59	5.34 ± 3.06	< 0.001
Ordered drugs per patient	9.37 ± 4.21	12.44 ± 4.24	7.53 ± 3.06	< 0.001

Descriptive statistics including central tendency measures and variability measures were used to describe the data. Kolmogorov-Smirnov test was used to evaluate the normal distribution of the variables. Independent sample t-test, One-way Analysis of Variance, and chi-square test were applied to assess differences among groups, where appropriate. Pearson correlation coefficient was used to assess the relationship between quantitative variables. Multivariate linear and logistic regression was applied with to predict dependent variables including age, gender, type of cancer, source of cancer, number of ordered drugs and length of hospital stay based on independent variables including having at least one potential DDI on the phamacotherapeutic protocol, and number of potential DDIs per patients. Odds ratios and their 95% confidence intervals (CI) were estimated through logistic regression model. Probability value of less than 0.05 was considered significant.

## Results

For one year period from July 2009 to July 2010, chart records’ drugs orders of 224 consecutive eligible cancer hospitalized patients were studied. Patients’ characteristics are shown in Table 3. Median age of patients was 50 years ranging from 14 to 90 years. Females were 37.5% of patients and approximately 73% of patients were diagnosed with a solid tumor. Length of hospital stay for patients varied from 2 to 40 days, with a median of 5 days. Number of drugs in patients order ranged from 2 to 24 drugs, with a median of 8 drugs.

Number of potential DDIs found for each patient in the ward ranged from 0-19 interactions and the mean of potential DDI for each patient is 1.02. All potential for these 224 patients were 228 interaction in this one year and 84 patients (37.5%, 95%CI: 31-44%) had at least one potential interaction on their screening.

Severity and documentation levels of potential DDIs are shown on Tables 4 and 5. According to the severity level, 14.03% of interactions were classified as major and 59.65% of them classified as moderate. Based on the documentation level, 19.29% of the interactions classified as established and probable.

A qualitative study was done on the screened potential DDIs. The most frequent combination with the potential of interaction in 224 patients was Cisplatin with Furosemide which encountered 19 times. Next potential interaction found was Magnesium hydroxide with Dexamethasone which encountered 7 times. Potential interaction of Magnesium hydroxide with Ranitidine encountered 6 times. Potential interactions between Ciprofloxacin with Doxorubicin and Acetaminophen with Furosemide had the next frequency by 5 times.

Table 6 demonstrates characteristics of the two groups of patient: patients with no potential drug interaction and patients which have at least one potential interaction on their orders. Patients with at least one potential interaction are likely to be older than 61 years old than patients with no potential drug interaction (p = 0.024). But no significant association explored between patients older than 61 years old and number of interactions and also no correlation was evident between number of interactions in patients and age (r = - 0.05, p = 0.938). Gender has no significant tendency on two groups of patients, with and without potential interaction, and also gender did not affect number of potential DDIs per patients. In patient with hematologic cancer having potential DDI was significantly more comparing with patients with a solid tumor (54.09% versus 31.28%) (p = 0.002) and also number of interactions in patients with hematologic cancer is significantly more than number of interactions in patients with solid tumor (p = 0.019). Number of ordered drugs and longer length of hospital stay were associated with having potential DDI in patients (p < 0.001, p < 0.001). Also both number of the ordered drugs and longer length of hospital stay were correlated with the number of interactions found for each patients (r = 0.601 and 0.806, p < 0.001 and p < 0.001). Source of cancer in different organs have also associated with having potential DDI in patients (p < 0.001). All patients with esophageal cancer, testicular cancer and cervical cancer have at least one interaction on their medications. So these patients are more probable to have interactions. Also number of interactions per patient depends on the source of cancer. (p = 0.007).

Multivariate analysis showed that being older than 61 years old, suffering from hematologic cancer, source of cancer in different specific organs (esophagus, testis and cervices more than other sources), and number of ordered drugs for patients were independent predictors of having at least one potential DDI in hospital order.

Multivariate analysis on number of interactions per patients as an independent factor shows that suffering from hematologic cancer, source of cancer in different organs, length of hospital stay and number of ordered drugs for patients were independent predictors for this variable.

## Discussion

Cancer patients are at the high risk of drug-drug interactions due to their complex pharamacotherapeutical medications. Becoming hospitalized after a complication increases the number of medications and raises the risk of interactions on these people. It has been shown that 4% mortality in hospitalized oncology patients is because of their medication DDIs (9).

Frequency of potential DDIs and the risk factors has widely been investigated in hospital of modern countries (7-14) but it has not been considered lot in developing countries. In a Canadian study (2007), the frequency of potential drug interactions between outpatients receiving systemic anticancer therapy for solid tumors in a 4 weeks period was 27%. (10) In this study we want to encounter the frequency of potential DDIs in a hematology-oncology ward in a developing country.

Although all medical orders through hospital stay have been screened for each patient, frequency of potential drug interactions was 37.5% of patients. In a study in Brazil (2005), unrelated to chemotherapy, potential of DDIs for 100 in-hospital cancer patients found to be 63% in the midpoint of hospital stay but this study didn’t investigated all patients’ medication.

In this study, on the qualitative analysis, nearly 28% of all interactions were for cancer medications interactions with each other or with a complication pharmacotherapy. Cisplatin and Furosemide screened as about 30% of these cancer medication interaction. Cisplatin can increase the ototoxicity effects of loop diuretics and hearing tests is necessary when administering them together (16). Cyclophosphamide founded to have 9 different interactions which was the most number between cancer medications, which can be due to the usage of this drug for different types of malignancy. Nearly 22% of these interactions were for co-administration for Ciprofloxacin with Vincristine, Doxorubicin and cytarabine which must be considered more in cancer patients, because patients with leukemia and lymphoma are more susceptible to infection during and after chemotherapy, so they use many antibiotics such as ciprofloxacin. Some important interactions in these cases are shown in Table 7. On non-chemotherapy medications co-administration of Magnesium Hydroxide with Dexamethasone, Ranitidine, Furosemide and Acetaminophen have the most frequency. Receiving chemotherapy regimen, cancer patients are at the risk of gastrointestinal adverse effects like dyspepsia, diarrhea, constipation and reflux. Since the more antacid they use for these adverse effects, the more shown interactions will happens. So it is recommended to use the Proton-pump inhibitors such as pantoprazol (not omeprazole) instead of antacids or use lactulose as a laxative instead of magnesium hydroxide which may have less DDIs. Some important interactions between non-chemotherapy medications in these cases are shown in Table 8.

**Table 7:** Some important interactions between cancer medications with each other and with other complications’ medication.

**Co-administration**	**Interaction**
Mercaptopurine and Allopurinol	Allopurinol increase pharmacologic and toxic effect of Mercaptopurine when orally administered.
Fluorouracil and Warfarin	Fluorouracil increase the anticoagulant effect of Warfarin
Methotrexate and Vancomycin	Vancomycin can increase the risk of Methotrexate toxicity by elevating its serum concentration and delaying its clearance
Methotrexate and Cotrimoxazole (TMP-SMZ)	MTX-induced bone marrow suppression can be increase by using Cotrimoxazole. Methotrexate also may predispose patients to (TMP-SMZ)-induces megaloblastic anemia
Irinotecan and Phenytoin	Phenytoin can reduce the antitumor activity of Irinotecan
Vincristine and Azole antifungal	Azole antifungals can increase the risk of Vinca alkaloid toxicity
Cyclophosphamide and warfarin	Cyclophosphamide can increase the anticoagulant effect of Warfarin
Etoposide and Warfarin	Etoposide can increase the anticoagulant effect of Warfarin

It must be cleared that these 37.5% interactions which found from all patients are potential drug-drug interactions which only screened through all patients medical orders at the length of hospital stay on the reference. As it can be seen nearly 10% of these interactions are just theoretical mechanisms of action and just about 10% of them confirmed by large clinical trials. It must be mentioned that the clinical incidence of the interactions are not followed in the ward. Although reviewing hospital records of patients may include the noting bias which was a limitation of study.

In conclusion having a DDI seems to be more likely to occur in patient olders than 61 years old. Hematologic cancers, having more medications in physician’s order, longer length of hospital stay, esophageal cancer, testicular cancer and cervical cancer have related to having a DDI and also having more number of interactions. In other studies increasing numbers of drugs, type of medication (drugs to treat comorbid conditions), presence of brain tumors found to be risk factors for patients interaction. (9) In Brazilian study which was a developing country (2005) being more than 67 years, hospital stay more than 6 days and having more than 8 drugs in order was the risk factor for DDI which is consistent with the results found in this study. It is important to consider these risk factors to recognize DDIs occurrence and minimize the dangerous effect of their incidence. It could be important to highlight the role of clinical pharmacists in having collaboration with oncologists on hematology and oncology wards of developing countries.
